# Hemiclonal analysis of interacting phenotypes in male and female *Drosophila melanogaster*

**DOI:** 10.1186/1471-2148-14-95

**Published:** 2014-05-03

**Authors:** Hannah ME Tennant, Erin E Sonser, Tristan AF Long

**Affiliations:** 1 Department of Biology, Wilfrid Laurier University, Waterloo, Ontario N2L 3C5, Canada

**Keywords:** Sexual selection, Mate choice, Female choosiness, Male attractiveness, Drosophila melanogaster, Hemiclonal analysis, Interacting phenotypes, Mating speed

## Abstract

**Background:**

Identifying the sources of variation in mating interactions between males and females is important because this variation influences the strength and/or the direction of sexual selection that populations experience. While the origins and effects of variation in male attractiveness and ornamentation have received much scrutiny, the causes and consequences of intraspecific variation in females have been relatively overlooked. We used cytogenetic cloning techniques developed for *Drosophila melanogaster* to create “hemiclonal” males and females with whom we directly observed sexual interaction between individuals of different known genetic backgrounds and measured subsequent reproductive outcomes. Using this approach, we were able to quantify the genetic contribution of each mate to the observed phenotypic variation in biologically important traits including mating speed, copulation duration, and subsequent offspring production, as well as measure the magnitude and direction of intersexual genetic correlation between female choosiness and male attractiveness.

**Results:**

We found significant additive genetic variation contributing to mating speed that can be attributed to male genetic identity, female genetic identity, but not their interaction. Furthermore we found that phenotypic variation in copulation duration had a significant male-associated genetic component. Female genetic identity and the interaction between male and female genetic identity accounted for a substantial amount of the observed phenotypic variation in egg size. Although previous research predicts a trade-off between egg size and fecundity, this was not evident in our results. We found a strong *negative* genetic correlation between female choosiness and male attractiveness, a result that suggests a potentially important role for sexually antagonistic alleles in sexual selection processes in our population.

**Conclusion:**

These results further our understanding of sexual selection because they identify that genetic identity plays a significant role in phenotypic variation in female behaviour and fecundity. This variation may be potentially due to ongoing sexual conflict found between the sexes for interacting phenotypes. Our unexpected observation of a negative correlation between female choosiness and male attractiveness highlights the need for more explicit theoretical models of genetic covariance to investigate the coevolution of female choosiness and male attractiveness.

## Background

Females often differ in their response to male courtship. This difference in female “responsiveness” (the likelihood that a female will respond to a potential mate) may be influenced by a number of factors including her prior mating experience, social experience, and environmental/developmental conditions [1,2]. Similarly, variation in female “choosiness” (the degree to which females discriminate amongst potential mates) may arise from the relative costs and benefits associated with female mate choice (i.e. time and energy costs) [2,3]. Theoretical and empirical work on sexual selection has shown considerable variation, both phenotypic and genetic, among females in their responses to sexually selected male traits [4]. Female responsiveness has been shown to exhibit additive genetic variation [5-7] and it is widely accepted that genetic variation in female choosiness is necessary for species to evolve via sexual selection [1-3]. However, despite its importance in understanding models of sexual selection, there is little information about the extent and nature of heritable genetic variation in female mating behaviours [6,7]. The difficulty in studying this suite of traits stems in part from the complexity of quantifying the genetic basis of female choosiness. Of the numerous empirical studies on variation in female choosiness [1,5-7], only a few have emerged with clear generalities about within population levels of genetic variation in female choosiness (see [4,8]). These studies often involve comparing females from genetically isolated populations [5,7], whereas investigating the sources of this variation *within* populations is ultimately important to understanding variation in female choosiness and its role as a selective force.

Variation in female choosiness may be attributed to “innate preferences” which reflect the heritable genetic component in sensory organ development [2]. For example, individual female guppies, *Poecilia reticulata,* may respond differently to male orange spots because of the level of sensitivity to that signal in the retina [9]. Female preference and the preferred male trait (the orange spot) are then maintained by sexual selection as they are coevolving through a positive genetic correlation [10]. Fisher’s runaway selection predicts a positive genetic correlation between female preference and male attractiveness, with the genetic correlation arising through pleiotropy or linkage disequilibrium [10-14]. Despite this predicted positive genetic correlation between female preference and male attractiveness, the ambiguity of empirical studies makes it hard to identify the sources of observed covariance [15]. Ultimately, variation in female choosiness can affect the strength, direction, and nature of sexual selection acting on sexually selected male traits (usually decreasing the overall strength), which can affect male courtship displays and, indirectly, the female's responses to them [1,2].

Not only can female responsiveness to male signals determine whether or not mating occurs, but it may be manifested in post-copulatory phenotypes, such as maternal investment patterns into offspring. In species that are polyandrous, a female might adaptively alter her investment strategy depending on the specific qualities (i.e. the direct/indirect costs and benefits) associated with her most recent mate in order to maximize her lifetime reproductive success [16]. According to the differential allocation hypothesis, differences in investment may be manifested in the total amount and/or quality of parental care provided, as well as by altering the number and/or size of offspring produced [17]. For example, female Australian Rainbow fish, *Melanotaenia australis*, will produce twice as many eggs when they mate with more “attractive” (i.e. larger) males than with less “attractive” (i.e. smaller) males [18]. Adjusting patterns of investment into offspring can have direct consequences for the future success of those offspring. For instance, in the fruit fly, *Drosophila melanogaster,* egg size is positively correlated with variance in egg hatchability, pre-adult size, juvenile survival, and adult starvation resistance [19], and as such may be strongly influenced by specific maternal investment strategies. Such investment strategies may differ between species depending on the patterns of parental care. For example, in species with bi-parental care, females are more likely to invest more into clutch size rather than egg size, simply due to the fact that a highly attractive male may signal high-quality parental care [20]. Conversely, in species which lack parental care maternal investment in egg size rather than egg number is likely, often to compensate for poor egg viability [20].

Although there is considerable evidence supporting differences in allocation in relation to phenotypic traits of males (such as body size, male ornamentation, etc.) [18,21-24], there is scant evidence regarding whether there is genetic variation for this ability in females. Recently, an attempt was made to address this issue by measuring differences in allocation in assays where the genetic identity of male *D. melanogaster* was experimentally varied across numerous mating pairs [25]. It was found that male genotype appeared to influence both the number and size of the eggs produced after a mating. Additionally, a negative trade-off between female fecundity and egg size was also demonstrated, consistent with earlier findings [26]. However, in this experiment, the genetic identity of all the females was uniform; thus the potential for female genetic identity and the interaction between males’ genotypes with *different* females was not explored. Thus, only a fraction of the total genetic variation for any interacting phenotype may be determined when testing each sex independently, potentially ignoring genetic contributions from the mating partner as well as interactions between both individuals’ genotypes [27]. There is increasing evidence that phenotypic traits in one individual may be influenced by another individual’s genotype [27,28]; these effects are known as indirect genetic effects (IGEs). IGEs likely modify genetic architecture, therefore resulting in genetic variance components in interactions between conspecifics [27]. Hemiclonal analysis [29,30] allows us to partition out the effect a conspecific genotype has on another individual’s genotype.

Previous work on genetic variation in female preference has primarily focused on varying the genetic identity of one sex (typically the male) and holding female genetic identity static [4,6,25,31]. To our knowledge, no previous study has examined female choosiness (the degree to which females discriminate among potential mates), female responsiveness (the likelihood a female will respond to a potential mate), *and* maternal investment patterns while simultaneously varying *both* male and female genetic identity. Additionally, studies examining the genetic covariance between female choosiness and male attractiveness are mixed; some have found a transient positive correlation that disappears after one generation of random mating [6,13,32], others have found no correlation at all [15,33,34]. While the prediction of a positive genetic correlation between male attractiveness and female choosiness is a central element of Fisherian runaway selection [10] it is not essential to other models of sexual selection. For instance, sensory bias [35] does not predict any particular genetic correlation between male attractiveness and female choosiness, leading many to incorrectly assume that in the absence of a genetic correlation, sensory bias must be occurring [35,36]. No other models (indirect benefits [37], good genes [38], or sexual conflict [39]) depend on a positive genetic correlation and have been modelled without any correlation between female choosiness and male attractiveness. Interestingly, other models, such as sexual conflict, might predict a negative genetic correlation between female choosiness and male attractiveness due to interlocus sexual conflict between sex-specific fitness optimizing strategies [40-42]. Further empirical estimates of genetic correlation may allow for clearer interpretations of models in order to make better predictions for how species evolve via sexual selection.

In this study we set out to investigate the roles of male and female genetic identity on mating behaviour in *Drosophila melanogaster*; a species with a polyandrous mating system where males do not provide any obvious post-fertilization parental care [43]. By creating hemiclonal lines, we are able to investigate the causes and consequences of genetic variation in both pre- and post-copulatory traits, using two aspects of female preference: female choosiness *sensu*[1,44] and female responsiveness *sensu*[4]. From measurements of females’ behaviours, we are able to quantify female choosiness, female responsiveness, male attractiveness, female investment into her offspring, and determine how these phenotypes are related to her genotype, the genotype of her mate, and the interaction between them.

## Results

### Partitioning of variance: genetic identity and pre-copulatory interacting phenotypes

Of a total of 1967 pairs of flies that were observed, 1667 pairs initiated copulation within the 90 min observation time frame. For all possible male–female mating combinations we have data on the proportion of pairs that successfully mated, including the latency to mating, and the copulation duration for these successful mating pairs. We decided to exclude those mating pairs from subsequent analysis as we did not want to inflate our estimate of variance components. This did not have any effect on the analyses of our results, as non-mating was randomly distributed across all mating pairs so that excluding them was not statistically biasing any combination (χ^2^ = 126; p = 0.32). If we included those non-mating pairs (substituted a value of 90 min for mating latency – the maximum duration of observation), we found, for the most part, the same results as in our more conservative data set. Using an REML approach we were able to quantify the extent to which phenotypic variation in mating speed was dependent on genetic identity of one or both sexes. We found a small, but significant amount of the variance in mating speed could be attributed to differences in female genetic identity (7.96%) and to differences in male genetic identity (7.56%), but there was no statistically detectable interaction between the two (Table 1). Copulation duration (CD) also varied between the 12 hemiclone lines (Table 1). Male genetic identity had a significant effect on the amount of CD variance (4.06%), while female genetic identity accounted for a non-significant 1.75% of the observed variation. The notable difference when including all non-mating pairs in the statistical analysis is a significant effect of male and female interaction on mating speed (5.1%; Additional file 1: Table S1).

**Table 1:** Decomposition of variance components of interacting phenotypes for 12 hemiclone lines using REML

**Interacting phenotype**	**Source of variation**	**Variance component**	**SE**	**95%lower**	**95%upper**	**% of total**
Mating speed	Female	18.9	4.86	9.32	28.48	7.96
	Male	17.97	4.65	8.85	27.09	7.56
	Female x Male	0.95	5.95	-10.72	12.63	0.4
	Residual	199.71	8.84	183.44	218.25	84.07
	Total	237.54				100.00
Copulation duration	Female	0.43	0.25	-0.05	0.91	1.75
	Male	0.99	0.34	0.32	1.66	4.06
	Female x Male	-0.02	0.67	-1.32	1.29	-0.07
	Residual	23.07	1.01	21.21	25.18	94.27
	Total	24.47				100.00
Number of eggs laid in 1^st^ 24 hrs	Female	0.31	0.07	0.16	0.45	12.18
	Male	-0.01	0.02	-0.05	0.31	-0.33
	Female x Male	0.23	0.69	0.09	0.36	9.17
	Residual	1.99	0.87	1.83	2.17	78.98
	Total	2.52				100.00
Egg length	Female	5.6 × 10^-5^	1.5 × 10^-5^	2.6 × 10^-5^	8.6 × 10^-5^	8.15
	Male	-6.09 × 10^-6^	4.4 × 10^-6^	-1.5 × 10^-5^	2.5 × 10^-6^	0.00
	Female x Male	0.00017	1.6 × 10^-5^	0.00014	0.00020	25.29
	Residual	0.00046	8.1 × 10^-6^	0.00044	0.00047	66.56
	Total	0.00069				100.00
Egg width	Female	5.05 × 10^-6^	1.35 × 10^-6^	2.40 × 10^-6^	7.71 × 10^-6^	8.58
	Male	3.65 × 10^-7^	4.83 × 10^-7^	-5.82 × 10^-7^	1.31 × 10^-6^	0.62
	Female x Male	1.36 × 10^-5^	1.25 × 10^-6^	0.000011	1.61 × 10^-5^	23.18
	Residual	3.98 × 10^-5^	7.03 × 10^-7^	3.85 × 10^-5^	4.13 × 10^-5^	67.63
	Total	5.89 × 10^-5^				100.00
Egg volume	Female	7.3 × 10^-7^	1.4 × 10^-7^	4.5 × 10^-7^	1.0 × 10^-6^	40.40
	Male	1.0 × 10^-8^	1.3ex10^-8^	-1.6 × 10^-8^	3.6 × 10^-8^	0.55
	Female x Male	3.4 × 10^-7^	2.9 × 10^-8^	2.8 × 10^-7^	3.9 × 10^-7^	18.86
	Residual	7.2 × 10^-7^	1.3 × 10^-8^	6.9 × 10^-7^	7.5 × 10^-7^	40.18
	Total	1.8 × 10^-6^				100.00

### Partitioning of variance: genetic identity and post-copulatory interacting phenotypes

REML results (Table 1) indicated that female genetic identity (F) and the interaction between female and male identities (FxM) both accounted for a sizeable amount of the observed phenotypic variation in both egg length (F = 8.15%; FxM = 25.29%, Table 1) and width (F = 8.58%; FxM = 23.18%, Table 1). Similarly, female genetic identity accounted for 40.40% of the observed variation in egg volume and female x male genetic identities accounted for an additional 18.86% of the variance. The number of eggs laid in the first 24 hour period following the behavioural assay were significantly influenced by female genetic identity (17.67%, Table 1), the specific interaction of male and female genetic identities (6.13%), but not significantly by male genetic identity (0.94%).

### Trade-offs between fecundity and egg size

**Figure 1:**
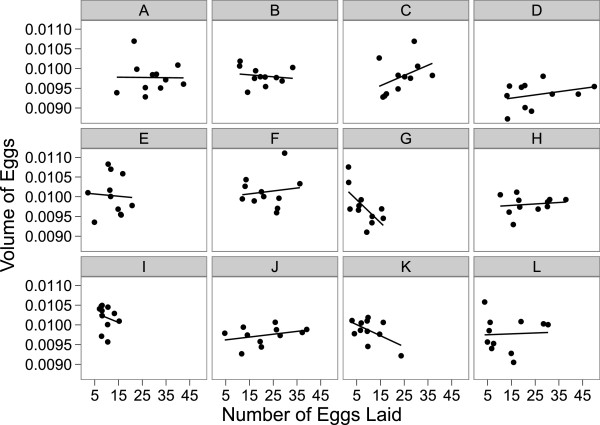
**Trade-off between egg size and egg number among 12 female hemiclone lines.** Individual plots each represent one female hemiclone line **(A-L)** and each point on the graph represents an average for both the number and volume of eggs laid when a hemiclone female mated with one of the 11 other male genotypes. Regression lines indicate only 2 of 12 female hemiclone lines **(G and I)** show a significant negative trade-off between egg volume and egg number.

**Figure 2:**
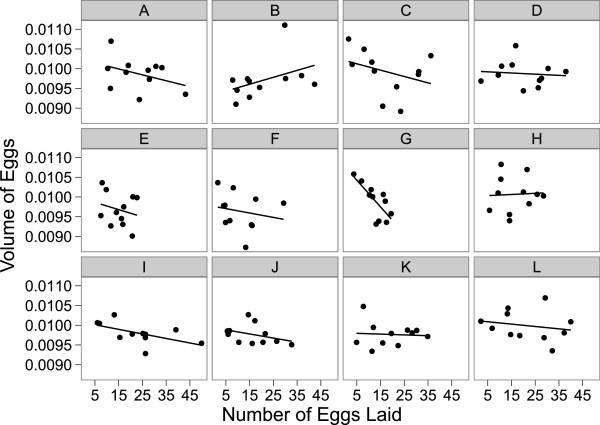
**Trade-off between egg size and egg number among 12 male hemiclone lines.** Individual plots each represent one male hemiclone line **(A-L)** and each point on the graph represents an average for both the number and volume of eggs laid when a hemiclone male mated with one of the 11 other female genotypes. Regression lines indicate only 1 of 12 male hemiclone lines **(G)** show a significant negative trade-off between egg volume and egg number.

By examining the relationship between the number of eggs and the size of eggs laid by each female hemiclone line when mated to males from the other 11 hemiclone lines we were able to look for evidence of trade-offs. Only 2 of the 12 female genotypes assayed exhibited a significant negative relationship, suggestive of a trade-off between egg size and number (Figure 1). Overall the mean of the 12 regression lines was not significantly different from zero ($\overset{¯}{x}$ =-5.585 × 10^-6^, t_11_ = 0.8801, p = 0.3976). Interestingly, the slope of the regression lines was more negative in hemiclone lines of low fecundity F_(1,10)_ = (13.42), corr = (0.76), p = (0.0044), slopes: G = (-5.81 × 10^-5^), I = (-2.44 × 10^-5^). Furthermore, we found that only 1 of the male genotypes exhibited a significant negative relationship (Figure 2) between female fecundity and egg size. The same significant male genotype also demonstrated the lowest fecundity.

### Genetic correlation between attractiveness and choosiness

**Figure 3:**
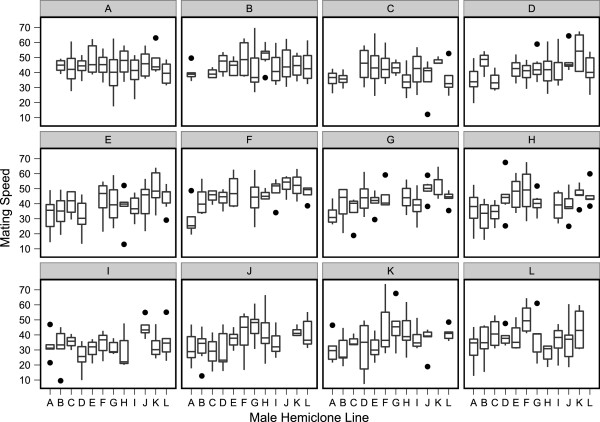
**Female responsiveness and female choosiness for male attractiveness.** Each individual plot represents data collected from one female hemiclone line for the time to mating with 11 different male hemiclones (excluding the intercrosses). Female responsiveness is measured as the mean mating speed among female hemiclone lines and is evident in the variation among lines in the height of the means. Female choosiness is measured as the variance of that mean (responsiveness) with the choosiest females having the most variance in responses. Differences in the height of mating speed indicate male attractiveness, i.e. the faster the mating speed (lower y-values), the more attractive the male. Male hemiclone lines are ordered from the most attractive **(A)** to the least attractive **(L)**, left to right, along the x- axis.

**Figure 4:**
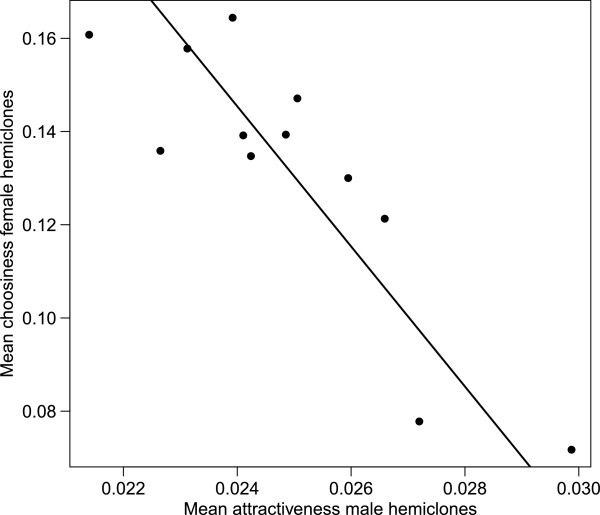
**A negative genetic correlation between male attractiveness and female choosiness.** We estimated genetic correlation by regressing mean male mating speed (attractiveness) on the coefficient of variance (CV) (choosiness) for all 12 hemiclone lines. This association indicates that the genotypes which produce highly attractive males also produce non-choosey females, and vice versa (P = 0.0006, r = -0.836, n = 12). We used the inverse of mean male mating speed to demonstrate the negative genetic correlation so that the larger x-values corresponded to attractive males.

From the variation in mean mating speed for each female hemiclone measured with each of her 11 possible hemiclone males (Figure 3), we calculated the coefficient of variance (CV) as an index of her degree of female choosiness [7]. The mean mating speed of each male hemiclone line (based on mating speed obtained with each of the other 11 female hemiclone lines) was used to calculate male attractiveness (with longer times to mate indicating “less attractive” males ([45]). Our estimates of female choosiness and male attractiveness between the two analyses (non-mating pairs included and excluded) are significantly positively correlated (female choosiness: t = 3.44, df = 11, p = 0.0063; male attractiveness: t = 10.26, df = 11, p = 0.0001). We examined the genetic correlation between the two variables and found a strong negative correlation between male attractiveness and female choosiness (r = -0.836, p = 0.0006, n = 12; Figure 4). The complete analysis including all non-mating pairs also demonstrates a significant negative correlation (r = -0.584, p = 0.0458, n = 12; Additional file 2: Figure S1). The haploid genome that produced the most choosey females also yielded the least attractive males, while the genotype producing the least choosey females yielded the most attractive males.

## Discussion

### Influence of genetic identity on pre-copulatory interacting phenotypes

The relationship between female preferences and male display traits is central to the function of inter-sexual selection and understanding the causes and consequences of its variation is of great importance to the fields of behavioural genetics and evolutionary biology [4]. Using hemiclonal analysis we clearly demonstrate the underlying genetic basis for variation in several interacting phenotypes (mating speed, copulation duration, and fecundity) present in this population and how these traits are affected by the genetic identity of each sex.

Differences in the specific genetic identity of males and females both individually (but not jointly) had a significant effect on the variation in mating speed. This indicates that females varied genetically in their receptivity to the available male, and males differed genetically in their attractiveness. However, a lack of a significant male x female interaction suggests that these factors acted independently of each other. Previously [8,31] it was found that female genotype (but not male genotype) strongly influenced the variance in mating speed, which is consistent with the theory that this trait is controlled primarily by the female [45]. This may also have been due to the willingness of females to mate simply because of an association with the ability to produce eggs, but no significant association between mating speed and female fecundity was found (t = -0.7373, df = 10, p = 0.4779; Additional file 3: Figure S2). Females also appeared to rank male phenotypes the same (i.e. females tended to “agree” on male attractiveness).

It was somewhat surprising that we found no evidence for significant male x female genotype interactions for mating speed, as previous work has demonstrated within population genetic variation for this trait in male and female *D. melanogaster*[46,47]. By mating males to two different female genotypes (low receptivity *vs.* high receptivity), it appeared that the expression of mating speed in both absolute and relative performance of male genotypes in *D. melanogaster* was strongly influenced by the female genotype [47]. In this study, the interaction between genotypes was so dramatic that a given male genotype could be among the quickest to mate with one female genotype, yet among the slowest when presented with another female genotype. In a similar study, the male x female genotype interaction contributed to 38.1% of the variance observed in mating speed, suggesting that the mating speed of males was strongly influenced by the genetic identity of the female they courted [31]. Therefore, variation in mating speed among females may be determined by female responsiveness, varying according to female genotype, and the effectiveness of male courtship may depend on the genotype of the female being courted. The significant MxF for mating speed from our estimates of variance components using the complete analysis is likely due to our data set, and not experimental design.

Compared to previous research, there may be some differences in the amount of genetic variation present in the current study system and those used by others [31,47]. For example, studies have used isofemale lines (inbred lines of the same population) and therefore have low genetic variation [48] and low potential for G x E interaction within isofemale lines. The covariance of interacting phenotypes may also be affected by relatedness of individuals. Relatedness produces a predictable covariance between phenotypes of interacting individuals [27]. Since related individuals share genes, a covariance is due to phenotypic similarity. In our assays we used hemiclonal analysis, which allows for genetic variation and natural selection to act on the male and female hemiclones [39], increasing the potential for GxE interactions. The use of different source populations of *D. melanogaster* can also strongly influence the composition of genetic variation present [31]. Genetic incompatibilities as a result of outbreeding may lead to variance in mating speed and other pre-copulatory traits. Differentially adapted genotypes can also result in low genetic diversity, as divided populations may have evolved different co-adapted gene complexes, resulting in reduced fitness of hybrids when individuals from different populations mate [49]. There is strong evidence that geographically distinct populations of *D. melanogaster* have genetic variation in pre-copulatory traits due to differences in selection history and genetic architecture [2,50] that may not be present within each population; future studies should consider this.

Male genotype significantly contributed to the amount of variation in copulation duration, a result which is consistent with theory and previous evidence that this trait is primarily under male control [51]. Increasing the duration of copulation may potentially be associated with direct fitness benefits for males, (i.e. ensuring paternity in competitive environments), via transfer of increased number of sperm in the presence of rival males [52], and/or transferring products that are (indirectly) harmful to females by reducing their lifespan [53,54], subsequent reproductive success [53-55], and female remating rate [56]. Reducing the risk of sperm competition by prolonged copulation duration allows males to achieve high fertilization success [51].

We found no significant interaction between male and female genetic identities for phenotypic variation in copulation duration. Previous work also reported no significant interaction between male and female genotypes in *D. mojavensis*, suggesting that genotypic differences did not account for behavioural interactions [57]. This is somewhat surprising since recent studies have determined that females exert at least some control over copulation duration in *Drosophila* species [58-60]*.* It may be in the best interest for both sexes that sperm transfer is successful because both individuals have made the choice to mate with each other. A lack of a significant interaction between male and female genotype suggests that there may be limited opportunity for coevolution for copulation duration [61], male and female *D. melanogaster* may be dealing with different suites of traits associated with copulation duration, or selection pressures may differ between the sexes for this trait, varying copulation duration optima [40,62]. From the male’s perspective, selection may favour longer copulation for transferring accessory seminal proteins (Acps), increasing the likelihood of siring a female’s clutch [51,55] and succeeding in sperm competition [63] (although factors other than copulation duration may contribute to the allocation of Acps [64]). On the other hand, females may suffer physical harm during copulation [65] and/or the contents of male ejaculate may be detrimental to female fitness (see below), thus selection may favour shorter copulation. Further investigation of copulation duration as an interacting phenotype and whether or not it is subject to sexual selection is warranted.

### A negative correlation between female choosiness and male attractiveness

We found a significant negative genetic correlation between female choosiness and male attractiveness. This association indicates that the genotypes which produce highly attractive males also produce females of low choosiness, and vice versa. According to predictions of the Fisherian model of sexual selection, a positive genetic correlation between male attractiveness and female choosiness would result in both attractive males and choosey females [2,3,8,12]. While previous empirical tests of genetic correlations between male attractiveness and female choosiness have yielded mixed results (see [6,7,15,34,66]), this is, to the best of our knowledge, the first instance where a negative correlation has been reported. Instead our results show that the production of choosey female genotypes also yields unattractive male genotypes, and vice versa, consistent with sexual conflict theory [40,42,67]. Our negative correlation may reflect the effect of sexually antagonistic genetic variation in our population.

The adaptive benefit of female choosiness is a component of almost all models of sexual selection – whereby females exhibiting non-random mating patterns gain a direct and/or indirect fitness advantage [1,3]. It follows, therefore, that females of high fitness would be more choosey than those that were less choosey, and that the two traits should be positively genetically correlated. Similarly, the evolution of elaborate display traits in males is viewed as being adaptive, as those who possess them are viewed as more attractive, and will be at a selective advantage in acquiring mates and/or post-copulatory success [3]. However, it is becoming increasingly evident that the fitness maximizing strategies of males and females are often incompatible, and traits that increase fitness in one sex, decrease fitness in the other sex [40,62]. This sexual conflict can arise either via the evolution of antagonistic adaptations in males and females under sex-specific expression (interlocus sexual conflict) or on traits with a common genetic basis in both sexes (intralocus sexual conflict) [68-70]. One of the consequences of intra locus sexual conflict is that the fitness consequences of alleles will depend on the sexual genetic background in which it is expressed. Genotypes resulting in high male fitness will yield low female fitness (and vice versa) [40,57]. Here, we suggest that the presence of sexually-antagonistic alleles in our laboratory population (a common observation in *D. melanogaster* stocks – see [40,62]) may be the root cause of our observed negative genetic correlation between female choosiness and male attractiveness. As stated above, each of these traits is likely to be genetically correlated with fitness-related traits (in their respective sexes), and if some of these fitness-related traits have a genetic architecture that is the subject of intra locus sexual conflict, then as a result, female choosiness and male attractiveness will ultimately show a negative genetic correlation.

Whether or not this pattern is limited to our laboratory population or may be more widespread is unclear and is deserving of further investigation. However, there is increasing evidence that traits (and fitness) in wild populations show the signs of being subject to genetic tug-of-war between the sexes [68,69]. Furthermore, the absence of many clear examples of positive genetic correlations between choosiness and attractiveness may be in part due to a wide-spread role of this co-evolutionary conflict. Our experimental results will hopefully stimulate theoretical models to further consider the implications of negative genetic correlations in shaping species’ evolutionary trajectories via sexual selection.

### Trade-offs between fecundity and egg size

Our examination of a potential trade-off between egg provisioning and production found that only 2 out of 12 female hemiclone lines surveyed displayed a significant negative relationship between fecundity and average egg size. When viewed from the male hemiclone perspective, only 1 genotype out of 12 exhibited a significant negative trade-off, suggesting that males were able to influence females similarly in egg production and provisioning, possibly due to experimental design (lack of male-male competition, no-choice assay). Genetic models of life history evolution predict a negative correlation between egg size and fecundity [26], and thus it is of interest to investigate the reasons why the majority of hemiclone females did not show a trade-off between fecundity and egg volume.

A negative correlation between egg size and egg number is expected when clutch size (=egg volume x egg number) is constant [71], and a change in egg size is associated with a concomitant change in egg number [26]. The lack of a relationship suggests that the phenotypic trade-off between egg size and number may evolve independently without a direct genetic trade-off [26]. Non-significant correlations between egg size and number may also be due to variation in reproductive investment between male and female genotypes, and physical condition. Since environmental conditions and resource availability were constant for all aspects of our study, we can probably rule out environmental variation as a factor (trade-offs allow a female to optimize fitness by maximizing resource potential [72]; when resources are in abundance, a trade-off may not exist (see [71,73]). Reproductive investment often increases with female body size [71,74,75]. Larger females are predicted to produce more eggs, therefore the fitness gain in terms of eggs fertilized will be greater in large females [75,76] than with small females of low fecundity [77,78]. Natural variation in female body size could influence clutch size and result in large variation in egg number, therefore producing non-negative correlations between egg size and number [71].

Genetic variation among female genotypes in the provisioning and production of eggs and genetic variation among male genotypes in their ability to stimulate both egg production and provisioning in females could lead to differences in clutch size. The use of hemiclonal lines allowed us to create many individuals of a consistent haplotype expressed in either a male or a female genetic background in an outbred state [30]. Cross-mating these individuals enabled us to examine the effect of *both* maternal and paternal genotype, while also considering sex-specific effects within and among hemiclone lines. Depending on the female genotype, certain male genotypes may only be successful in stimulating either egg size or female fecundity in their mates, but not both traits simultaneously. Attractive males may stimulate short-term female fecundity by transferring accessory seminal proteins (Acps) in the ejaculate to females during copulation. These Acps stimulate oogenesis and ovulation in females after mating when there is sperm available to fertilize the eggs, increasing the egg laying rate [78]. Males differ genetically in their stimulatory capacity towards females [79] and females vary genetically in their seminal receptors [56,78]. This is reflected in our REML analysis which shows a significant interaction between male and female genotypes in terms of female fecundity and egg size.

Sexual conflict theory predicts that there is genetic variation among males for harm imposed upon females and genetic variation among females for resistance to males [80], which is consistent with the theory of sexually antagonistic coevolution [81]. Female *D. melanogaster* suffer direct costs when mated with attractive males [82], and may attempt to reduce these costs by “resisting” copulation with attractive (and presumably harmful) males [83]. Females stimulated into mating with attractive males have an increased short-term fecundity, but decreased overall lifetime reproductive success [55,84], whereas females stimulated into mating with unattractive males may suffer immediate fitness costs, but benefit long term by reduced personal harm and potentially higher quality offspring [85]. The effect of male harm to females is reflected in female egg laying patterns. In *D. melanogaster,* large males are presumed to be more attractive because they may be better at stimulating/coercing potential mates [53,54,86]. The larger the male, the bigger the accessory glands [86,87], and thus the more Acps can potentially be transferred in the ejaculate during copulation, depending on female mating status and the risk of sperm competition [64,88]. However, in addition to boosting female short-term fecundity, Acps also reduce female longevity [82], alter feeding behaviour [89], and induce a refractory period [76,82]. Choosey females who avoid mating with harmful males may resist the negative effects of male courtship via better control over their own reproductive physiology. By “controlling” who they mate with (i.e. avoiding the largest, most attractive males via pre-copulatory mate choice [85]), these females may mediate the dosage of short-term fecundity-stimulating seminal fluid they receive, resulting in lower short-term fecundity [54]. Non-choosey females may be unable to resist/distinguish harmful (attractive) males as effectively as choosey females, resulting in an increase in their short-term fecundity [53,54,82].

We did not see a consistent significant relationship between provisioning and production of eggs when varying both parental genotypes (in contrast to previous studies varying only male genetic identity [25]). Our study suggests that these patterns are a result of a female’s genetic identity, and not necessarily dependent on her mate. Our results also demonstrate how genotype x genotype interactions and resource availability may play a significant role in maternal investment patterns.

### Influence of parental genotype on egg size and number

In *D. melanogaster,* both male and female genotype influenced the number and size of eggs produced from mating pairs. Using an REML approach we were able to determine that ~60% of the observed phenotypic variation seen in egg size could be collectively attributed to the genetic identities of one (the female) or both of the individuals in a mating pair (Table 1). Female genotype accounted for the largest amount of the variation seen in egg size. As mentioned previously, egg size can be a proxy of female maternal investment strategies and is important to the future success of offspring in many animals [90]. Offspring genotype may play a role in determining nutrient usage as maternal investment nutrient-wise can be a limiting factor for offspring development [90]. Studies of maternal effects have shown that maternal genotype accounts for approximately half of the variance in offspring phenotype [91] while the direct effect of the offspring’s genotype accounts for between 10-50% of the phenotypic variance [91], suggesting that paternal genotype may also influence offspring phenotypic variance. This creates a “multi-layered” indirect genetic effect (IGE) wherein the maternal genotype’s “environment” is influenced by variation in the paternal genotype, subsequently influencing the fitness variance in future offspring [27,28].

We found significant differences in egg size variation due to the interaction of male and female genetic identity, suggesting that some contribution from the ejaculate may influence egg production. Some contents of a male’s ejaculate may be allocated as nutrients for the eggs e.g. [92], or more importantly, act as stimulants for egg production/investment [55,76] resulting in various egg sizes (i.e. females who receive larger amounts of seminal product may lay larger eggs than those females who receive less [90]). In *D. melanogaster*, larger eggs have higher viability and greater successful larval development rates [19], therefore it is of interest to both the male and female that offspring viability is successful. However, since the interactions of male and female genotypes had such a significant effect on egg size, this highlights the importance for *both* males and females to be choosey in their mate selection.

Female genotype significantly influenced the number of eggs laid after 24 hours post-mating, suggesting that females vary genetically in their oviposition rates [93]. A significant interaction between male and female genotypes for this trait suggests that females also differ genetically in response to male seminal products [93]. The number of eggs sired by a male may be due to the composition and/or amount of his ejaculate which might reflect differences in types and/or amounts of components. Since accessory protein composition exhibits genetic variation among males in *D. melanogaster* for oogenesis and oviposition stimulation [81], females may not only differ in responsiveness, but may receive different kinds of bioactive components from male ejaculate to incorporate into their eggs [90] resulting in variation in the number of eggs laid. Male accessory proteins may also affect female behaviour and physiology by increasing the rate of eggs produced, resulting in a short-term increase in the number of eggs laid [79,94]. This would also increase male reproductive success, suggesting that it may rely on both male and female genotype.

Male genetic identity alone did not account for a significant amount of the variation seen in egg size or egg number. The eggs measured in our study represented the females’ 2^nd^ clutch (see Methods), and therefore developed in the presence of male seminal products. Males may benefit female fecundity in the short-term by transferring accessory seminal proteins (Acps) to females during mating [79]. These Acps stimulate oogenesis and ovulation in females after mating when there is sperm available to fertilize the eggs, increasing the egg laying rate [76]. Variation in egg size and number in a female’s 2^nd^ clutch attributed to male genotype has been found [25], suggesting that a male’s genotype influences a female’s fecundity and the size of eggs she produces. However, only the effects of male genotype on maternal investment patterns were previously tested as the genetic identity of the females was held constant, limiting their ability to draw conclusions about the effects of both parental identities on maternal investment patterns or their interactions [25]. Our results suggest that the interaction of genetic identity plays a significant role in maternal investment patterns, as females from the same hemiclone line (i.e. carrying the same haploid genome, and therefore of similar size) invested differently when mated with different male hemiclonal lines.

## Conclusion

In conclusion, we demonstrated a genetic basis for variation in female choosiness and female responsiveness. When mated with non-related individuals, males and females differed genetically in their sexual responsiveness but did not differentially respond to their mate's genetic identity. We also discovered a strong negative correlation between female choosiness and male attractiveness. The combined genetic identities of mating pairs had a significant effect on the amount or quality of resources a female will invest into her offspring. The interaction of male and female genotypes influencing fecundity and/or offspring size can result in a coevolution between males and females for investment into reproductive success.

Our results indicate that whether or not sex-limited interacting phenotype development extinguishes intralocus sexual conflict may depend on a population’s genetic architecture and selective history [95]. Intralocus sexual conflict may be interfering with adaptive evolution in our population because of evidence that sexually antagonistic selection can lead to a trade-off between the optimal genotypes for males and females, biasing the reproductive outcome towards one sex, influencing the maintenance of genetic variation, and ultimately the evolutionary trajectory in a population. Our results confirming MxF genetic variation for mating speed and maternal investment support the prediction that indirect genetic effects act on pre- and post-copulatory traits in *D. melanogaster*.

Further studies on the plasticity of female choosiness, body size, and the correlation between choosiness and lifetime reproductive success could offer insight into whether or not condition-dependence influences genetic variation in the interacting phenotypes studied. More empirical studies investigating genotype x genotype interactions in genetically different individuals for both pre- and post-copulatory behaviours should support the above findings.

## Methods

### Experimental populations

The ultimate source of the genetic variation in our assays were *D. melanogaster* obtained from the *Ives* (hereafter “IV”) population; a large, (N ~ 5600 adults), outbred wild-type population initially derived from South Amherst, MA, USA in 1975, which has been maintained under standardized culture condition since 1980 [96]. The IV population has previously been shown to exhibit considerable genetic variation for a variety of adult life history traits [97,98]. This population, like all others used in this assay, is maintained in vials on a discrete 14-day culture cycle. Flies are reared at a controlled density (~100 eggs per vial), on a banana/agar/killed-yeast medium at 25°C, with a 12L:12D diurnal light cycle. A replicate population, IV-*bw*, is maintained under similar conditions and was created by repeatedly backcrossing the recessive brown-eyed allele, *bw-*, into the IV genetic background for 10 consecutive generations. Subsequent backcrossing is periodically done to ensure the IV-*bw* population is sound.

### Hemiclonal analysis

In order to determine whether phenotypic variation in pre and post-copulatory behaviours could be attributable to additive genetic variation in males and/or females, we used a hemiclonal analysis approach (see [29,30]). This quantitative genetic technique is available in *D. melanogaster* due to a natural lack of recombination in males of this species, and the availability of phenotypically-marked artificial cytogenetic constructs (described below), which together can be used to isolate, replicate and propagate nearly-complete haploid genomes (for details see [30,40]). These cloned haploid genomes can then be expressed in a “hemiclonal” state in either a male or a female genetic background (consisting of a random sample of wild-type haplotypes sampled from the base IV population). This technique has been used to quantify genetic variation in a variety of behavioural and morphological traits [30] but has never before been used to explore female mate choice or egg production.

For this assay, we randomly chose 12 clone lines from a larger collection of 31 that had been sampled from the IV population in May 2012. Each clone line is propagated with the use of females from a “clone-generator” population [99], who possess a random Y chromosome, a conjoined “double X” chromosome [*C*(1)*DX*, *y*, *f*], and are homozygous for translocated autosomes [T(2;3) *rdg*C *st in ri p*^P^ bw^D^]. Creation of male hemiclones was obtained by mating clone males to virgin females from a population (“DX-IV”) possessing the “double-X” chromosome, but otherwise possess a random sample of autosomes originating from the IV population. Creation of hemiclonal females involved mating clone males to virgin females obtained from the IV population. Many of the eggs produced via these crosses are not viable due to chromosomal imbalances (50% mortality of eggs laid by IV females mated to clone males, and 75% mortality of eggs laid by DX-IV females mated to clone males). As larval density has important consequences for adult phenotypes and life histories [100] great care was taken to ensure that the developmental conditions of vials containing developing hemiclones resembled the conditions typically experienced in the IV population. Thus, we added eggs (of the same age) from the IV-*bw* population to each of our experimental hemiclone-producing vials in order to ensure a desirable density of 100 viable larvae per vial. Specifically, each vial that would yield male hemiclones received 100 eggs laid by clone-mated DX-IV females, and 75 IV-*bw* eggs, while each vial that would yield female hemiclones received 100 eggs laid by clone-mated IV females, and 50 IV-*bw* eggs. These vials were then reared under standard environmental conditions. Nine days later, wild-type virgin hemiclonal females were collected within 6 hours of eclosion from their pupae. Wild-type male hemiclones were collected on the 11^th^ day, to ensure they had experience courting receptive females [101]. All hemiclones were kept in individual vials prior to the mating assay, which was conducted on the 13^th^ day of the flies’ life (i.e. 3–4 days post-eclosion).

### Behavioural assays

Standard no-choice preference tests (see [34,102]) were conducted to conveniently measure a female’s latency to mating when placed with a single male as an indication of male attractiveness and avoid the potential confounds of male-male competition. Since we were primarily interested in global male attractiveness, rather than what trait(s) were preferred, we measured *all* traits that confer male attractiveness [44,103]. Additionally, we point out that identical outcomes were found when assessing female preference in both choice *vs*. no-choice experiments using other species of *Drosophila*[104,105], but to our knowledge none have been done with *D. melanogaster*. An individual non-virgin hemiclone male was placed in a vial with an individual virgin hemiclone female from a different hemiclone line. This was repeated for all 12 lines, resulting in 132/144 possible combinations of individual mating pairs (excluding the intercrosses), with 3 replicates per block, resulting in a total of 396 vials to observe. We deliberately avoided creating crosses where males and females were of the same hemiclone origin because there is evidence that related individuals may behave differently in mate preference than between unrelated mating pairs (see [49]).

Assays began at 9:00 am EST, which corresponds to the time when the incubator lights turn on, and flies become sexually active (H.T. Obsv). Assays were run in the same environmentally controlled room where the flies were cultured and stored prior to the assay. We recorded the date and time for each assay to control for any experimental block effects, which were then accounted for in statistical analysis (see below).

Female responsiveness was quantified using the mean mating speed (or latency to copulation, including courtship) and was measured as the time the vials from each female hemiclone line were placed in view of the observer to the moment copulation began. Since all female genotypes were exposed to essentially the same 11 multiple male genotypes (because of excluded intercrosses) acceptance of a male by female after taking time to assess potential mates reflected female choosiness. Thus, female choosiness was quantified as the standard deviation in female responsiveness across male hemiclone line (see statistical analysis). Male attractiveness was defined as the average responsiveness for each female genotype to the 11 other male genotypes (*sensu*[8]). Quantifying all phenotypes influencing male attractiveness allowed us to determine whether or not male attractiveness has a genetic basis. Copulation duration was measured as the time the male mounted the female to when the pair disentangled. Each individual mating pair was observed for a period of 90 minutes until copulation was observed. If copulation was ongoing at the 90 minute mark, the mating pair was observed until copulation ended. Our conservative analysis excluded any non-mating pairs, where our complete analysis reflected the latency to mating as 90 min.

### Measurement of maternal investment: volume and number of eggs laid

Immediately following the preference assays, all males were removed from the vials using light CO_2_ anesthesia. The vials containing only females were placed in the incubator for 24 hours to allow the females to lay eggs. The next morning, the number of eggs laid by each female were counted using a stereo light microscope to determine any immediate post-copulatory effects of male genetic identity on fecundity. At this time, the 3 females from the replicate crosses were placed together into a small egg laying chamber outfitted with a disc of coloured media [106], and left to lay eggs for an additional 24 hours, as the effects of males on egg size may not be detectable until 24 hours after mating occurs [25]. The following morning, all of the chambers were immediately placed into the refrigerator for 24 h to ensure there were no changes in egg sizes due to further egg development. A pilot study confirmed that this short-term refrigeration had no significant effect on egg size measurements (E. Sonser, unpublished data). Upon retrieval from the refrigerator, the eggs that had been laid were counted and then photographed using a microscope-mounted camera. All eggs were placed in the same orientation (i.e. ventrally or dorsally; not laterally) to control for any variation in measurements that could arise from different orientations. ObjectJ (Vischer & Nastasa, University of Amsterdam), a plug in for ImageJ 1.46n (Rasband, National Institute for Mental Health), was used to measure the eggs’ lengths and widths to the nearest thousandth of a millimeter. Length was defined as the measurement of the polar axis, while the width was the diameter of the egg, orthogonal to the length and at the widest point. From these values, the volume of the eggs was calculated using the formula for a prolate spheroid: V = 1/6πW^2^L (as per [25,107,108]). From previous studies [108] it is known that there is considerable variation in egg volume as well as in length and width, which is why it is important to consider absolute size (i.e. volume) when investigating maternal investment patterns. Repeatability scores were calculated for measurements of both egg length (96%) and egg width (91%) indicating that one measurement per egg would give us precise measurements.

### Statistical analysis

Statistical analysis was completed using JMP 8.0.0 (SAS Institute, Cary, NC) and R version 2.13.1 (The R Foundation for Statistical Computing) to determine the role of genetic identity in *D. melanogaster* mating behaviours. Sources of variation in behavioural, morphological, and fecundity data were analyzed using a restricted maximum likelihood (REML) approach because it gave an accurate estimate of variance components when sample sizes were not perfectly balanced [109]. The genetic variation for mating speed, copulation duration, egg length, and egg width was estimated using a random effects variance component estimate. Female genetic identity, male genetic identity, and the interaction of male and female genetic identities were nested within experimental block and modelled as random effects. Mating speed and copulation duration was square root transformed to obtain normality of distributions and differences in average blocks was accounted for (as in [79]) by multiplying data from each block by the inverse of the ratio of the block mean to the global mean across all blocks. To estimate the additive genetic variation seen among all 12 of our hemiclone lines we partitioned the variance of mating speed, copulation duration, and egg size for block effect, male genetic identity, female genetic identity, and the interaction of the two. Significance was determined by examining the lower 95% confidence interval of the estimate to see if it included zero. Data for non-mating pairs was excluded from this statistical analysis.

To represent genetic variance in female responsiveness, female responsiveness was measured as the mean mating speed of each female hemiclone line across mean male hemiclone lines. Since mating speed is thought to be controlled primarily by female genotype [45], this variable was used to quantify male attractiveness (i.e. average response of female genotype to the male genotype).

To determine the genetic correlation between male attractiveness and female choosiness we followed established procedures [5-7]. Female choosiness was calculated as the coefficient of variance (CV) and was obtained by calculating the standard deviation of the mean mating speed for female hemiclone lines (calculated by obtaining the mean mating speed value for each female hemiclone line mated with each male hemiclone line and averaged across experimental block) [7]. To ensure independence of male and female genotypes (which could cause a positive correlation by influencing the x and y values) the experiment did not include intercrosses between males and females of the same hemiclone line. We then regressed female choosiness on male global attractiveness for all 12 hemiclone lines.

To determine if any trade-off existed between provisioning (i.e. egg size) and production (i.e. egg number) we performed correlation tests and plotted regression lines representing the relationship between provisioning and production for each female hemiclone line. Data for non-mating pairs was excluded from all statistical analyses (except see Results).

## Competing interests

The authors declare that they have no competing interests.

## Authors’ contributions

HMET and TAFL conceived of the study. All authors helped design and conduct the experiment. TAFL performed the statistical analyses. HMET and EES drafted the manuscript. All authors read and approved the final manuscript.

## Supplementary Material

Additional file 1: Table S1 Inclusive estimates of variance components of mating speed for 12 hemiclone lines using REML. Click here for file

Additional file 2: Figure S1 A negative genetic correlation between male attractiveness and female choosiness. Our estimates of female choosiness and male attractiveness incorporated non-mating pairs with a latency of 90mins. This association indicates that the genotypes which produce highly attractive males also produce non-choosey females, and vice versa (P = 0.0006, r = -0.836, n = 12). We used the inverse of mean male mating speed to demonstrate the negative genetic correlation so that the larger x-values corresponded to attractive males. Click here for file

Additional file 3: Figure S2 No correlation between latency to mating and female fecundity. We estimated the correlation between latency to mating and female fecundity for each of the 12 female hemiclone lines (t = -0.7373, df = 11, p = 0.4779). The phenotypic variation for female mating speed was not due to an association between female’s willingness to mate and the ability to produce eggs. Click here for file
