# Supplementary material for: Hemiclonal analysis of interacting phenotypes in male and female Drosophila melanogaster
Source: BMC Evol Biol. 2014 May 3;14:95. doi: 10.1186/1471-2148-14-95 (PMC4101844; doi:10.1186/1471-2148-14-95)
Supplement: Additional file 1: Table S1 — Inclusive estimates of variance components of mating speed for 12 hemiclone lines using REML. [file 1471-2148-14-95-S1.pdf]

Additional file 1: Table S1: Inclusive estimates of variance components of mating speed for 12 hemiclone lines using REML.

Interacting phenotype	Source of variation	Variance component	SE	95% Lower	95% Upper	% of Total
Mating speed	Female	15.70	4.89	6.10	25.28	4.53
	Male	22.42	6.00	10.66	34.19	6.48
	Female x Male	17.65	8.17	1.63	33.65	5.10
	Residual	290.80				83.19
	Total	346.56				100.00
