# Supplementary figures and images for: Hemiclonal analysis of interacting phenotypes in male and female Drosophila melanogaster
Source: BMC Evol Biol. 2014 May 3;14:95. doi: 10.1186/1471-2148-14-95 (PMC4101844; doi:10.1186/1471-2148-14-95)

**Additional file 2: Figure S1**

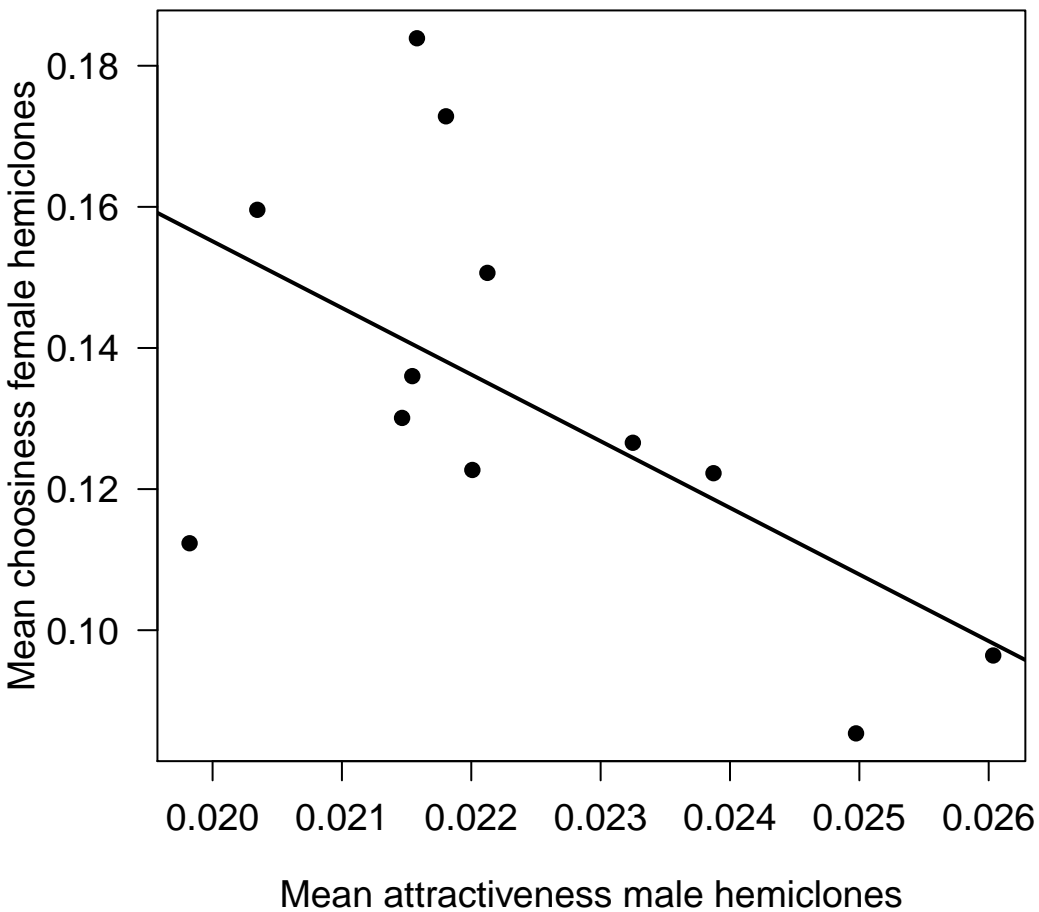

Supplement: Additional file 2: Figure S1 — A negative genetic correlation between male attractiveness and female choosiness. Our estimates of female choosiness and male attractiveness incorporated non-mating pairs with a latency of 90mins. This association indicates that the genotypes which produce highly attractive males also produce non-choosey females, and vice versa (P = 0.0006, r = -0.836, n = 12). We used the inverse of mean male mating speed to demonstrate the negative genetic correlation so that the larger x-values corresponded to attractive males. [file 1471-2148-14-95-S2.pdf]

**Additional file 3: Figure S2**

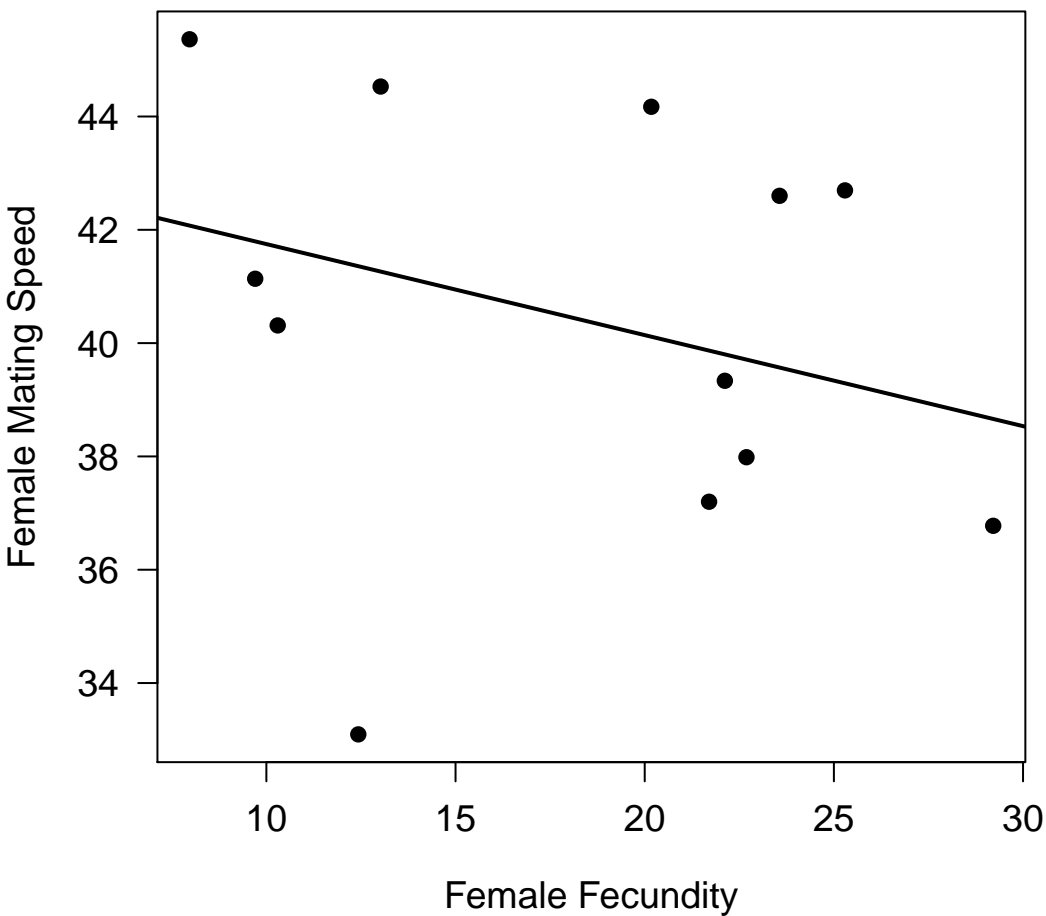

Supplement: Additional file 3: Figure S2 — No correlation between latency to mating and female fecundity. We estimated the correlation between latency to mating and female fecundity for each of the 12 female hemiclone lines (t = -0.7373, df = 11, p = 0.4779). The phenotypic variation for female mating speed was not due to an association between female’s willingness to mate and the ability to produce eggs. [file 1471-2148-14-95-S3.pdf]
